# Emotion dysregulation as a transdiagnostic mechanism of opioid misuse and suicidality among chronic pain patients

**DOI:** 10.1186/s40479-018-0088-6

**Published:** 2018-06-06

**Authors:** Michael R. Riquino, Sarah E. Priddy, Matthew O. Howard, Eric L. Garland

**Affiliations:** 1 University of Utah College of Social Work, 395 South 1500 East, Salt Lake City, UT 84112 USA; 2 Center on Mindfulness and Integrative Health Intervention Development, 395 South 1500 East, Salt Lake City, UT 84112 USA; 3 University of North Carolina at Chapel Hill, Tate Turner Kuralt Building, Chapel Hill, NC 25799 USA

**Keywords:** Anhedonia, Chronic pain, Emotion dysregulation, Opioid misuse, Reinforcement, Suicidality

## Abstract

**Background:**

Chronic pain is a prevalent condition that causes functional impairment and emotional suffering. To allay pain-induced suffering, opioids are often prescribed for chronic pain management. Yet, chronic pain patients on opioid therapy are at heightened risk for opioid misuse—behaviors that can lead to addiction and overdose. Relatedly, chronic pain patients are at elevated risk for suicidal ideation and suicidal behaviors.

**Main body:**

Opioid misuse and suicidality are maladaptive processes aimed at alleviating the negative emotional hyperreactivity, hedonic hyporeactivity, and emotion dysregulation experienced by chronic pain patients on opioid therapy. In this review, we explore the role of emotion dysregulation in chronic pain. We then describe why emotionally dysregulated chronic pain patients are vulnerable to opioid misuse and suicidality in response to these negative affective states.

**Conclusion:**

Emotion dysregulation is an important and malleable treatment target with the potential to reduce or prevent opioid misuse and suicidality among opioid-treated chronic pain patients.

## Background

Approximately 100 million Americans suffer from chronic pain, a condition compounded by maladaptive cognitive and emotional processes that often cooccur with protracted and severe physical suffering [1]. Opioid therapy continues to be the primary medical treatment for chronic pain despite associated risks, including opioid misuse, addiction, and overdose [2], as well as deleterious neuropsychopharmacologic effects of prolonged opioid exposure, including dysregulation in brain circuits undergirding reward processing, stress reactivity, and the proactive regulation of emotions [3, 4]. Given the magnitude of the current opioid crisis in the U.S., there is an urgent need to understand the psychological factors that drive individuals with chronic pain to misuse their prescribed opioid medications. Furthermore, the clinical presentation of opioid-treated chronic pain patients is often complicated by comorbid psychiatric distress, substance use disorders, and suicidality [5–7]—so-called “epidemics of despair” that account for rising mortality rates in the U.S. [8]—in part through intentional opioid overdose. Yet, biobehavioral mechanistic models linking chronic pain to opioid misuse and suicidal behavior are lacking.

In this conceptual review, we posit that the transdiagnostic process of emotion dysregulation is central to understanding why opioid-treated chronic pain patients engage in opioid misuse and suicidal behaviors. Although emotion dysregulation may result in multiple forms of maladaptive behavior, here we focus on opioid misuse and suicidality as sequelae of chronic pain due to their high prevalence and significant public health impact. Recent meta-analyses suggest that 25% of chronic pain patients engage in opioid-misusing behaviors like opioid dose escalation or self-medicating negative affective states with opioids [9]. As will be discussed later, opioid misuse is associated with adverse consequences such as increased sensitivity to pain and stress, decreased sensitivity to natural rewards, functional impairments, and overdose risk [10–12]. Similarly, suicidality, which encompasses both suicidal ideation and behaviors, is especially common among individuals with chronic pain. Chronic pain patients have nearly two times the risk of death by suicide and are two to three times more likely than individuals without chronic pain to report suicidal ideation or make suicide attempts [13]. Drug overdose is the most commonly reported means of attempting suicide among chronic pain patients [14]. Given high rates of suicidal ideation and suicide attempts among chronic pain patients, and the ready presence of lethal means via opioid prescription, risk of death by suicide warrants particular attention among opioid-treated chronic pain patients—a population already at increased mortality risk [15, 16].

The primary aim of this review is to explore interrelationships of emotion dysregulation and opioid-treated chronic pain, and in particular, to propose how these factors give rise to opioid misuse and suicidality. To this end, we first describe emotion dysregulation among opioid-treated chronic pain patients. We then explore the role of emotion dysregulation as a transdiagnostic process underlying the development and maintenance of opioid misuse and suicidality in this high-risk population. Finally, we conclude by considering how psychological interventions designed to enhance affect regulation might address the emotion dysregulatory processes subserving opioid misuse and suicidality among chronic pain patients receiving long-term opioid treatment.

## Emotion, appraisal, and emotion dysregulation in chronic pain

The cognitive-motivational-relational theory of emotion asserts that emotions arise in response to a cognitive appraisal of the meaning or significance of a particular stimulus context [17]. In other words, the relational meaning one derives through the process of appraisal in response to a given situation determines whether one experiences sadness, happiness, or some other emotion. From this perspective, appraisals drive emotions and thereby shape emotion regulatory attempts [17]. With respect to chronic pain, when patients experience an aggravation in the symptomatic expression of their underlying painful condition or encounter an emotionally-distressing situation, they may respond in maladaptive ways depending on how they appraise the situation. For example, appraisals of situational helplessness, hopelessness, or feelings of interpersonal burdensomeness may drive opioid misuse as a coping strategy or thoughts of suicide as a way to escape the situation.

**Fig. 1:**
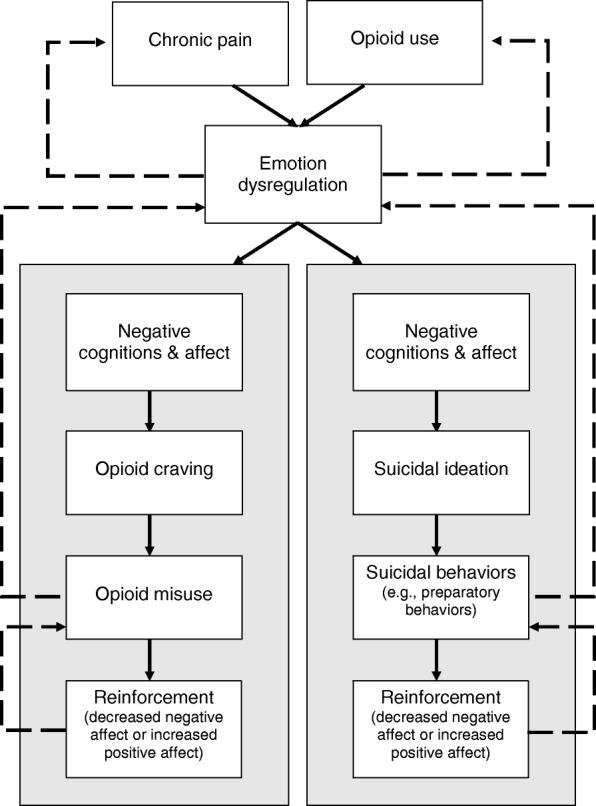
This model highlights the links between emotion dysregulation, opioid misuse, and suicide risk among chronic pain patients as outlined in this review. The recurrent experience of pain and long-term opioid exposure may drive emotion dysregulation in the form of negative emotional hyperreactivity and hedonic hyporeactivity, as well as deficits in the ability to proactively regulate emotions. Chronic pain patients prescribed long-term opioids who experience emotion dysregulation may respond with risky or maladaptive behaviors through a process of negative cognitions and affect. Specifically, as chronic pain patients become caught up in negative thoughts and feelings about their pain (e.g., pain catastrophizing), they may experience craving for opioids as a way to relieve those negative thoughts and feelings or thoughts of suicide as a way of escaping their present experiences. If they engage in opioid-misusing behaviors and experience either relief from negative affect or increased positive affect, they become more likely to engage in those behaviors as ways to manage distress through a process of reinforcement. Relatedly, suicidal behaviors, such as planning or preparatory behaviors, may result in relief from negative affect or increased positive affect when chronic pain patients feel like they have the means to escape their pain and distress. For example, hoarding medications, an indication of opioid misuse, can also be considered a preparatory behavior given the primary method of suicide planning endorsed by chronic pain patients is medication overdose. These links likely represent recursive processes, e.g., just as chronic pain and opioid use lead to emotion dysregulation, so does emotion dysregulation likely contribute to increased pain and opioid use. Similarly, although opioid misuse and suicidal behaviors may be employed in response to emotion dysregulation, they ultimately may lead to more frequent instances of emotion dysregulation

In this review, we posit that emotion dysregulation is the process that links these situational appraisals to maladaptive behaviors, including opioid misuse and suicidality (see Fig. 1 for a depiction of this process). Emotion *dysregulation* is marked by difficulties with the emotion-generative process and/or emotion regulation failures—i.e., not effectively employing adaptive emotion regulation strategies when it would be appropriate to do so [18]. The decreased hedonic capacity [19] and heightened stress sensitization [20] associated with chronic pain and long-term opioid therapy indicate *difficulties in the emotion-generative process*. Emotion-generation difficulties may result in persistent negative affect that contributes to emotion dysregulation [18], and thus, these difficulties are relevant to the development and maintenance of opioid misuse and suicidality. Relying on maladaptive coping strategies to regulate negative cognitions and affect (like misuse of opioids to self-medicate dysphoric emotional states or attempting suicide to escape from emotional suffering) would be considered *emotion regulation failures*. Additionally, reappraisal and suppression, classic forms of emotion regulation, may go awry in response to chronic pain [21]—a subject we will address in our discussion of factors contributing to emotion dysregulation among opioid-treated chronic pain patients.

### Difficulties with the emotion-generative process

In their review of the roles of emotion and emotion regulation in psychopathology, Gross and Jazaieri (2014) outlined areas where individuals might experience emotion-related difficulties, e.g., emotion intensity and emotion duration [18]. These difficulties with the emotion-generative process are relevant to chronic pain patients given the high levels of psychiatric comorbidities present in this population. Hyperreactivity or hyporeactivity, i.e., exhibiting too large or too small an emotional response to a given situation, are indicative of problematic emotional intensity [22]. Among chronic pain patients, distressing situations may elicit negative emotional hyperreactivity [20], whereas rewarding situations may result in blunted positive emotional responses—i.e., hedonic hyporeactivity [19]. Just as the experience of pain may modulate emotional intensity [23], pain may also affect the duration of emotional experience. As the experience of pain transitions from an acute to a chronic condition, negative affective reactions may become more common and long-lasting due to increasingly catastrophic appraisals of pain [24].

In the context of recurrent negative emotionality and hedonic hyporeactivity, individuals may become hypervigilant for perturbations to their normative physiological state and negatively appraise their bodily condition as problematic. For chronic pain patients, momentary fluctuations in pain or other somatic states may be misinterpreted as dangerous or an indication of damage to the body rather than benign physiological sensations that do not signal harm [25]. These negative interpretations can lead to a cascade of negative emotions or catastrophic thinking that interferes with individuals’ ability to regulate emotional distress. Given evidence for interoceptive deficits among individuals with chronic pain [26, 27], chronic pain patients may struggle with differentiating pain sensations from the autonomic, visceral, and musculoskeletal changes evoked by negative emotions. The tendency to label pain as “awful,” “horrible,” or with other emotionally-laden descriptors suggests pain can become conflated with the emotional distress it creates [28]. Because interoceptive awareness facilitates emotion regulation [29], chronic pain patients with interoceptive deficits may be less able to discriminate pain from the physical sequelae of negative emotions, and therefore are less inclined (or able) to engage in proactive emotion regulation attempts.

### Factors contributing to emotion dysregulation

When chronic pain patients do attempt to regulate their emotions, ineffective strategies or skills deficits may impede their ability to experience emotional relief. For example, in response to negative appraisals, suppression may be employed in an attempt to regulate negative emotional responses. However, suppression of unwanted thoughts and emotions paradoxically leads to more intense emotional reactions as cognitive resources become exhausted [21]. Additionally, thought suppression is associated with greater pain severity, pain interference, and depressive symptoms among chronic pain patients with trauma histories [30], and increased opioid craving among chronic pain patients with depressive symptoms [31]. Rather than engaging in suppression, individuals may attempt to regulate their emotions through reappraisal, a cognitive emotion regulation strategy focused on decreasing negative affect by reinterpreting situations in more adaptive ways [32]. However, studies suggest that both chronic pain and opioid use interfere with reappraisal processes and decrease reappraisal efficacy [33, 34]. From a neurobiological perspective, emotion regulation failures may result from inefficient top-down prefrontal modulation of bottom-up limbic activation—a pattern of neural dysfunction that has been observed among chronic pain patients [35]. When suppression or reappraisal efforts fail, opioid-treated pain patients may turn to problematic emotion regulation strategies (i.e., opioid misuse or suicidal behavior) as means of experiencing relief from their negative cognitions and affect.

## Emotion dysregulation as a transdiagnostic process in chronic pain

Mental health professionals have traditionally relied on categorical descriptions to classify psychiatric disorders, in contrast to a newer transdiagnostic perspective that eschews discrete taxonomies to consider underlying and universal processes undergirding impaired functioning [36]. A transdiagnostic approach may more effectively characterize the maladaptive psychological conditions that commonly cooccur with chronic pain [37, 38]. For example, chronic pain patients are at increased risk for comorbid psychiatric disorders, including depression, trauma, and substance use disorders. Indeed, it has been estimated that up to 85% of chronic pain patients experience severe depression [39, 40], between 10 and 50% report a history of trauma [41], and 16% exhibit substance use disorders above and beyond misuse of prescription opioids [7]. Although these psychiatric comorbidities have been traditionally studied as separate nosological entities with distinct etiologies, emerging research suggests that they share common transdiagnostic processes that may explain their association with chronic pain.

Emotion dysregulation is one such transdiagnostic process that subserves the development and maintenance of disrupted functioning and maladaptive behaviors across an array of physical and mental health conditions [42, 43]. Emotion dysregulation may be antecedent and consequent to chronic pain. For instance, individuals with a trait-like propensity towards emotion dysregulation may be at greater risk for developing chronic pain following an acute injury. However, as pain progresses from an acute to chronic condition, many individuals develop an attentional bias to pain-related information such that their attention becomes preferentially allocated to sensations of pain and environmental stimuli associated with pain episodes [44, 45]. Over time, the experience of pain coupled with this attentional bias can result in pain catastrophizing [46] and persistent negative cognitions, e.g., “This pain will never end” or “This is the worst pain I have ever felt.” When pain sensations are interpreted through the lens of catastrophic cognitive appraisals, chronic pain patients may experience dysregulated emotions, manifested by decreased distress tolerance [47] and heightened stress reactivity [20]. In turn, the consequent deficits in positive affect and surfeits in negative affect result in heightened pain sensitivity [48], which thereby exacerbates and prolongs chronic pain. The escalation over time of this downward spiral of pain sensations, biased attention to pain cues, pain catastrophizing, and dysregulated emotions may drive chronic pain patients to misuse opioids [4] or attempt suicide as ways of responding to their overwhelming distress.

For the remainder of this review, we focus on opioid misuse and suicidality—two maladaptive processes that are especially prevalent and pernicious among individuals with chronic pain. Chronic pain patients prescribed opioid therapy may turn to opioid misuse and suicidality in response to instances of emotion dysregulation, especially in the absence of other available adaptive coping strategies [9, 13]. Insofar as skill and self-efficacy are necessary for effective implementation of emotion regulation strategies [18], psychotherapeutic interventions that provide affect regulation training may prevent opioid misuse and suicidal behavior in the context of chronic pain. As such, we conclude with a discussion of promising behavioral treatment approaches to remediate emotion dysregulation among chronic pain patients [49, 50].

## Maladaptive responses to emotion dysregulation

### Opioid misuse among opioid-treated chronic pain patients

Despite increasing recognition of the public health risks of opioid analgesic pharmacotherapy [2], a dearth of accessible, alternative treatments has led to an over-reliance on opioids and adverse consequences for chronic pain patients [51]. These consequences include misuse of medication (e.g., unauthorized dose escalation) as a way to escape from pain, manage mood, and relieve stress—behaviors that are maintained through a process of negative reinforcement. Chronic pain is an ever-present, aversive experience. Research supports that relief of ongoing pain increases dopamine transmission and negative reinforcement of the behavior (e.g., [50, 51]). When a chronic pain patient relieves pain with opioids, taking medication is negatively reinforced [52, 53]. Over time, some patients will begin to take their medication more frequently or in higher doses to experience less pain or greater relief for longer periods of time. Neuropharmacological studies demonstrate that opioids bind to mu-opioid receptors in brain regions subserving pain perception, emotional experience, and reward (i.e., pleasure and well-being) [54]. Opioidergic activation of reward circuitry, including the ventral tegmental area and nucleus accumbens, results in feelings of euphoria [55]. Consequently, chronic pain patients begin to associate opioid-related cues (e.g., the sight of a pill bottle) with that euphoria [56], leading to an attentional bias toward opioid cues [57] and the subjective experience of craving [58] irrespective of the need to obtain pain relief. This process of conditioning may drive opioid dose escalation and result in opioid use that increases risk of overdose [54].

Moreover, chronic opioid use and misuse may result in allostatic changes to limbic and striatal brain circuitry leading to a persistent and escalating hedonic deficit characterized by increased sensitivity to stress and pain coupled with decreased sensitization to natural rewards [4, 59, 60]. As a result of this allostatic process, opioid-misusing chronic pain patients evince blunted autonomic responses during processing of natural rewards and while attempting to regulate negative emotions through reappraisal [61]. Indeed, opioid-misusing chronic pain patients report less use of reappraisal than pain patients who take opioids as prescribed, and these deficits in reappraisal use predict heightened affective distress and opioid craving (Garland EL, Hanley AW, Bedford C, Zubieta JK, Howard MO, Nakamura Y, Donaldson GW, Froeliger, B. Reappraisal deficits among prescription opioid misusing chronic pain patients, submitted). This opioid-induced emotion dysregulation may exacerbate pre-existing affective disorders that antedate chronic pain and initiation of opioid analgesic use [62, 63]. Thus, individuals who are vulnerable to negative affect may be more likely to misuse medication to offset dysphoria, which may exacerbate negative mood via allostatic neuroadaptations to brain circuits involved in emotion regulation. Relatedly, through processes of positive and negative reinforcement, individuals become more likely to continue engaging in opioid misuse as an emotion regulatory (i.e., coping) mechanism; the euphorigenic neuropsychopharmacological effects of opioids may temporarily increase positive affect and decrease negative affect, propelling the cycle of escalating opioid misuse toward opioid addiction [4].

### Suicidality among opioid-treated chronic pain patients

Although the mechanisms linking suicidality to chronic pain are still being explored, consistent evidence has demonstrated that chronic pain patients are at heightened risk for experiencing suicidal thoughts and exhibiting life-threatening behaviors [13, 64, 65]. The link between chronic pain and suicidality may, in part, be explained by emotion dysregulation. For example, suicidality is often preceded by persistent negative affect and anhedonia [66–69], two characteristics that often develop in response to pain. Specifically, as individuals experience repeated instances of heightened negative affect because of pain and stress (i.e., negative emotional hyperreactivity), they may experience a concomitant blunting of hedonic capacity—the ability to experience pleasure from naturally rewarding objects and events in the social environment [19]. This deficit in hedonic capacity may be exacerbated by the neuropsychopharmacologic effects of chronic opioid use, as articulated above. In the absence of healthy hedonic tone (i.e., hedonic hyporeactivity), chronic pain patients may respond to distressing situations with suicidal ideation, e.g., thoughts of escaping the resultant negative cognitive-affective states. In support of this, our team recently published a study on the association between suicidal ideation and prescription opioid craving and cue-reactivity [69]. Among a sample of 115 chronic pain patients, we found that suicidal ideation predicted opioid cue-reactivity, as measured by heart rate variability while completing a dot-probe task, via self-medication urges. Just as opioid misuse is strengthened through processes of negative and positive reinforcement, chronic pain patients may experience suicidality with increasing frequency and intensity due to physical pain and emotional distress. Suicidality is negatively reinforced insofar as suicidal ideation and suicidal behaviors (e.g., preparatory behaviors) may relieve negative cognitions and affect [70, 71]—in other words, as individuals consider suicide as a way of escaping pain or hoard their opioids in preparation of attempting suicide, they may experience relief from thoughts of burdensomeness or feelings of hopelessness now that they have determined a way to end their suffering. These suicidal thoughts and behaviors may produce positive affect, such as feelings of calm or acceptance of death. The dangerous escalation and cooccurrence of opioid misuse and suicidality may result in death by overdose or suicide if left untreated.

## Psychotherapeutic mechanisms addressing emotion dysregulation

A growing body of literature has illustrated the effectiveness of psychosocial interventions to address chronic pain. For example, a recent systematic review of randomized controlled trials (RCTs) of mindfulness-based interventions (MBIs) for chronic pain demonstrated significant improvements in pain, depression symptoms, and quality of life [50]. Similarly, cognitive-behavior therapy (CBT) has been extensively researched as a treatment for chronic pain, and has demonstrated efficacy for reducing pain-related interference by restructuring the cognitive distortions that arise in response to pain, as well as by increasing activity scheduling and pacing [72]. Though both MBIs and CBT can reduce negative emotional hyperreactivity [50, 72], neither MBIs nor CBT are specifically focused on remediating hedonic hyporeactivity undergirding opioid misuse and suicidality among chronic pain patients—a key risk mechanism thought to perpetuate the downward spiral of behavioral escalation articulated above. In contrast, Mindfulness-Oriented Recovery Enhancement (MORE) is a novel intervention that combines principles of mindfulness, CBT, and positive psychology to target hedonic dysregulation in addiction, affective disorders, and chronic pain through training in mindfulness, reappraisal, and savoring skills [73, 74]. Completed and ongoing RCTs are demonstrating positive effects of this intervention on treating pain symptoms and opioid misuse among chronic pain patients prescribed opioid therapy [75]. From a mechanistic perspective, the three primary components of MORE may be especially efficacious means of remediating the emotion dysregulation that impels both opioid misuse and suicidality among chronic pain patients.

Mindfulness can be conceptualized as a *practice*, a *state*, and a *trait* [76]. The state of mindfulness is characterized by a nonreactive, metacognitive awareness and acceptance of present moment thoughts, emotions, and sensations [77]. Mindfulness practices include mindful breathing, body scan meditations, and the informal practice of mindfulness during everyday tasks and activities. As one evokes the state of mindfulness through these practices, one begins to develop the trait of mindfulness, or dispositional mindfulness [78]. With respect to the focus of this article, trait mindfulness is positively associated with psychological well-being [79] and negatively associated with self-medication of negative emotions with opioids among a sample of chronic pain patients [80]. Mindfulness practice appears to strengthen the function and structure of prefrontally-mediated cognitive control networks [80–83], including those associated with emotion regulation, which in turn promotes top-down regulation of bottom-up emotional impulses [83, 84]. Moreover, mindfulness alleviates pain by facilitating a shift from affective to sensory processing of pain sensations [75, 85] and reducing thalamic amplification of nociceptive input via prefrontal cognitive control mechanisms [86, 87]. In these ways, mindfulness training can promote emotion regulation, reduce pain, and prevent maladaptive coping behaviors.

Reappraisal is an adaptive emotion regulation skill that can interrupt intense or persistent negative emotions [88]. Recent neuroscientific research demonstrates that it may also activate brain reward circuitry in ways consistent with positive emotion regulation [89]. Specifically, as individuals examine irrational or unhelpful cognitions that arise in response to distressing situations and recognize how those perceptions influence their emotional experiences, they can dispute those negative cognitions through the process of reappraisal and thereby experience consequent decreases in negative affect and increases in positive affect. Further, cognitive regulation strategies like reappraisal have been shown to be an especially potent means of decreasing pain intensity, in part though cortical modulation of pain and concomitant emotional reactivity [90, 91]. According to recent theorizing, reappraisal may also be strengthened by mindfulness practice [92]—a claim supported by empirical evidence [93, 94]. MORE capitalizes on the synergy of mindfulness and reappraisal to strengthen emotion regulation capacity.

The final component of MORE, savoring, targets hedonic hyporeactivity resulting from chronic pain and long-term exposure to opioids [60]. Savoring is an emotion regulation strategy in which the individual mindfully attends to features (e.g., the sight, sound, scent, and feel) of naturally-rewarding stimuli (e.g., beauty of the natural world, affiliative rewards, pleasurable physical sensations) while metacognitively monitoring and appreciating pleasant emotions and higher-order affective meaning arising from the encounter with the pleasant stimulus. According to the *restructuring reward hypothesis* [74], savoring may counter hedonic hyporeactivity underlying opioid misuse by shifting valuation of drug-related rewards back to valuation of natural rewards. A number of studies have provided support for the restructuring reward hypothesis by demonstrating effects of MORE on autonomic [95, 96], electrocortical [97], and neural functional [98] measures of reward processing that were in turn correlated with reductions in drug craving and use/misuse. Moreover, increasing positive affect has analgesic effects [99], and in that regard, increasing brain reward responses through mindfulness and other behavioral manipulations has been associated with decreased pain [100, 101].

MORE shares common transtherapeutic processes with other MBIs and CBT approaches; however, the unique integration of its three components may be especially effective for addressing emotion dysregulation among chronic pain patients. To be clear, whether or not they are combined in an integrative treatment package like MORE, therapeutic techniques involving mindfulness, reappraisal, and savoring can successfully treat the difficulties in emotion-generation and emotion regulation failures associated with maladaptive pain coping. Moreover, new affect regulation interventions and those that have demonstrated success in reducing suicidal ideation and substance misuse outside the context of chronic pain might be translated and tailored to address the unique clinical features of comorbid pain, suicidality, and opioid misuse.

## Conclusion

Cognitive, affective, and physiological antecedents and consequences of pain render chronic pain patients prescribed long-term opioid pharmacotherapy vulnerable to opioid misuse and suicidality—two hazardous behaviors with significant mortality risk. Established bidirectional relationships between pain, opioid use, and affective distress underscore the potential role of emotion dysregulation in the development and maintenance of opioid misuse and suicidality among chronic pain patients. As such, emotion dysregulation represents an important transdiagnostic treatment target for future prevention and intervention approaches designed to reduce lethal and life-threatening behaviors among opioid-treated chronic pain patients.

## Abbreviations

CBT: Cognitive-Behavior Therapy
MBI: Mindfulness-Based Intervention
MORE: Mindfulness-Oriented Recovery Enhancement

## References

[1] Dzau VJ, Pizzo PA (2014). Relieving pain in America: insights from an Institute of Medicine committee. J Am Med Assoc.

[2] Chou R, Deyo R, Devine B, Hansen R, Sullivan S, Jarvik J (2014). The effectiveness and risks of long-term opioid treatment of chronic pain: Evidence report/technology assessment.

[3] Elvemo NA, Landrø NI, Borchgrevink PC, Håberg AK (2015). Reward responsiveness in patients with chronic pain. Eur J Pain Lond Engl.

[4] Garland EL, Froeliger B, Zeiden F, Partin K, Howard MO (2013). The downward spiral of chronic pain, prescription opioid misuse, and addiction: cognitive, affective, and neuropsychopharmacologic pathways. Neurosci Biobehav Rev.

[5] Bosco MA, Gallinati JL, Clark ME. Conceptualizing and treating comorbid chronic pain and PTSD. Pain Res Treat. 2013;2013, 174728 10.1155/2013/174728 PMC3684116 23819047

[6] Bryant RA, O’Donnell ML, Creamer M, McFarlane AC, Clark CR, Silove D (2010). The psychiatric sequelae of traumatic injury. Am J Psychiatry.

[7] Manchikanti L, Cash KA, Damron KS, Manchukonda R, Pampati V, McManus C (2006). Controlled substance abuse and illicity drug use in chronic pain patients: an evaluation of multiple variables. Pain Physician.

[8] Case A, Deaton A (2015). Rising morbidity and mortality in midlife among white non-Hispanic Americans in the 21st century. Proc Natl Acad Sci.

[9] Vowles KE, McEntee ML, Julnes PS, Frohe T, Ney JP, van der Goes DN (2015). Rates of opioid misuse, abuse, and addiction in chronic pain: a systematic review and data synthesis. Pain.

[10] Volkow ND, Koob GF, McLellan AT (2016). Neurobiologic advances from the brain disease model of addiction. N Engl J Med.

[11] Atluri S, Sudarshan G, Manchikanti L (2014). Assessment of the trends in medical use and misuse of opioid analgesics from 2004 to 2011. Pain Physician..

[12] Martell B, O’Conner P, Kerns R, Becker W, Morales K, Kosten T (2007). Opioid treatment for chronic back pain: prevalence, efficacy, and association with addiction. Ann Intern Med.

[13] Tang NKY, Crane C (2006). Suicidality in chronic pain: a review of the prevalence, risk factors and psychological links. Psychol Med.

[14] Smith MT, Edwards RR, Robinson RC, Dworkin RH (2004). Suicidal ideation, plans, and attempts in chronic pain patients: factors associated with increased risk. Pain.

[15] Gomes T, Mamdani MM, Dhalla IA, Paterson JM, Juurlink DN (2011). Opioid dose and drug-related mortality in patients with nonmalignant pain. Arch Intern Med.

[16] Torrance N, Elliott AM, Lee AJ, Smith BH (2010). Severe chronic pain is associated with increased 10 year mortality. A cohort record linkage study. Eur J Pain.

[17] Lazarus RS (1993). From psychological distress to the emotions: a history of changing outlooks. Annu Rev Psychol.

[18] Gross JJ, Jazaieri H (2014). Emotion, emotion regulation, and psychopathology: an affective science perspective. Clin Psychol Sci.

[19] Borsook D, Linnman C, Faria V, Strassman AM, Becerra L, Elman I (2016). Reward deficiency and anti-reward in pain chronification. Neurosci Biobehav Rev.

[20] Vachon-Presseau E, Martel M-O, Roy M, Caron E, Albouy G, Marin M-F (2013). Acute stress contributes to individual differences in pain and pain-related brain activity in healthy and chronic pain patients. J Neurosci.

[21] Wenzlaff RM, Wegner DM (2000). Thought suppression. Annu Rev Psychol.

[22] Berenbaum H, Raghavan C, Le H-N, Vernon LL, Gomez JJA (2003). Taxonomy of emotional disturbances. Clin Psychol Sci Pract.

[23] Vachon-Presseau E, Roy M, Martel M-O, Caron E, Marin M-F, Chen J (2013). The stress model of chronic pain: evidence from basal cortisol and hippocampal structure and function in humans. Brain.

[24] Quartana PJ, Campbell CM, Edwards RR (2009). Pain catastrophizing: a critical review. Expert Rev Neurother.

[25] Heathcote LC, Jacobs K, Eccleston C, Fox E, Lau JYF (2017). Biased interpretations of ambiguous bodily threat information in adolescents with chronic pain. Pain.

[26] Lernia DD, Serino S, Riva G (2016). Pain in the body. Altered interoception in chronic pain conditions: a systematic review. Neurosci Biobehav Rev.

[27] Lernia DD, Serino S, Cipresso P, Riva G. Ghosts in the machine. Interoceptive modeling for chronic pain treatment. Front Neurosci. 2016;10(314). 10.3389/fnins.2016.00314 PMC4927564 27445681

[28] Arnow BA, Blasey CM, Constantino MJ, Robinson R, Hunkeler E, Lee J (2011). Catastrophizing, depression and pain-related disability. Gen Hosp Psychiatry.

[29] Fustos J, Gramann K, Herbert BM, Pollatos O (2012). On the embodiment of emotion regulation: interoceptive awareness facilitates reappraisal. SCAN.

[30] Pegram SE, Lumley MA, Jasinski MJ, Burns JW (2017). Psychological trauma exposure and pain-related outcomes among people with chronic low back pain: moderated mediation by thought suppression and social constraints. Ann Behav Med.

[31] Garland EL, Brown SM, Howard MO (2016). Thought suppression as a mediator of the association between depressed mood and prescription opioid craving among chronic pain patients. J Behav Med.

[32] Buhle JT, Silvers JA, Wager TD, Lopez R, Onyemekwu C, Kober H (2014). Cognitive reappraisal of emotion: a meta-analysis of human neuroimaging studies. Cereb Cortex.

[33] Lawrence JM, Hoeft F, Sheau KE, Mackey SC (2011). Strategy-dependent dissociation of the neural correlates involved in pain modulation. Anesthesiology.

[34] Mohajerin B, Dolatshahi B, Shahbaz AP, Farhoudian A (2013). Differences between expressive suppression and cognitive reappraisal in opioids and stimulant dependent patients. Int J High Risk Behav Addict.

[35] Ochsner KN, Ray RR, Hughes B, McRae K, Cooper JC, Weber J (2009). Bottom-up and top-down processes in emotion generation: common and distinct neural mechanisms. Psychol Sci.

[36] Garland EL, Howard MO (2014). A transdiagnostic perspective on cognitive, affective, and neurobiological processes underlying human suffering. Res Soc Work Pract.

[37] Crowe M, Whitehead L, Seaton P, Jordan J, Mccall C, Maskill V (2017). Qualitative meta-synthesis: the experience of chronic pain across conditions. J Adv Nurs.

[38] Linton SJ (2013). A transdiagnostic approach to pain and emotion. J Appl Biobehav Res.

[39] Bair MJ, Robinson RL, Katon W, Kroenke K. Depression and pain comorbidity: a literature review. Arch Intern Med. 2003;163(20):1587–9. 10.1001/archinte.163.20.2433 14609780

[40] Williams LS, Jones WJ, Shen JJ, Robinson RL, Weinberger M, Kroenke K (2003). Prevalence and impact of depression and pain in neurology outpatients. J Heurology Neurosurger Psychiatry.

[41] Fishbain DA, Pulikal A, Lewis JE, Gao J (2017). Chronic pain types differ in their reported prevalence of post-traumatic stress disorder (PTSD) and there is consistent evidence that chronic pain is associated with PTSD: an evidence-based structured systematic review. Pain Med.

[42] Fernandez KC, Jazaieri H, Gross JJ (2016). Emotion regulation: a transdiagnostic perspective on a new RDoC domain. Cogn Ther Res.

[43] Sloan E, Hall K, Moulding R, Bryce S, Mildred H, Staiger PK (2017). Emotion regulation as a transdiagnostic treatment construct across anxiety, depression, substance, eating and borderline personality disorders: a systematic review. Clin Psychol Rev.

[44] Schoth DE, Nunes VD, Liossi C (2012). Attentional bias towards pain-related information in chronic pain; a meta-analysis of visual-probe investigations. Clin Psychol Rev.

[45] Vlaeyen JWS, Morley S, Crombez G (2016). The experimental analysis of the interruptive, interfering, and identity-distorting effects of chronic pain. Behav Res Ther.

[46] Martel MO, Wasan AD, Jamison RN, Edwards RR (2013). Catastrophic thinking and increased risk for prescription opioid misuse in patients with chronic pain. Drug Alcohol Depend.

[47] McHugh RK, Weiss RD, Cornelius M, Martel MO, Jamison RN, Edwards RR (2016). Distress intolerance and prescription opioid misuse among patients with chronic pain. J Pain.

[48] Edwards RR, Dolman AJ, Michna E, Katz JN, Nedeljkovic SS, Janfaza D (2016). Changes in pain sensitivity and pain modulation during oral opioid treatment: the impact of negative affect. Pain Med.

[49] Goyal M, Singh S, Sibinga EMS (2014). Meditation programs for psychological stress and well-being. JAMA Intern Med.

[50] Hilton L, Hempel S, Ewing BA, Apaydin E, Xenakis L, Newberry S (2017). Mindfulness meditation for chronic pain: systematic review and meta-analysis. Ann Behav Med.

[51] Chou R, Turner JA, Devine EB, Hansen RN, Sullivan SD, Blazina I (2015). The effectiveness and risks of long-term opioid therapy for chronic pain: a systematic review for a National Institutes of Health pathways to prevention workshop. Ann Intern Med.

[52] Navratilova E, Xie JY, Okun A, Qu C, Eyde N, Ci S (2012). Pain relief produces negative reinforcement through activation of mesolimbic reward–valuation circuitry. Proc Natl Acad Sci.

[53] Xie JY, Qu C, Patwardhan A, Ossipov MH, Navratilova E, Becerra L (2014). Activation of mesocorticolimbic reward cirtcuits for assessment of relief of ongoing pain: a potential biomarker of efficacy. Pain.

[54] Volkow ND, McLellan AT (2016). Opioid abuse in chronic pain - misconceptions and mitigation strategies. N Engl J Med.

[55] Akil H, Watson SJ, Young E, Lewis ME, Khachaturian H, Walker JM (1984). Endogenous opioids: biology and function. Annu Rev Neurosci.

[56] Miguez G, Laborda MA, Miller RR (2014). Classical conditioning and pain: conditioned analgesia and hyperanalgesia. Acta Psychol.

[57] Garland EL, Froeliger BE, Passick SD, Howard MO (2013). Attentional bias for prescription opioid cues among opioid dependent chronic pain patients. J Behav Med.

[58] Ewan EE, Martin TJ (2013). Analgesics as reinforcers with chronic pain: evidence from operant studies. Neurosci Lett.

[59] Shurman J, Koob GF, Gutstein HB (2010). Opioids, pain, the brain, and hyperkatifeia: a framework for the rational use of opioids for pain. Pain Med Malden Mass.

[60] Elman I, Borsook D (2016). Common brain mechanisms of chronic pain and addiction. Neuron.

[61] Garland EL, Bryan CJ, Finan PH, Thomas EA, Priddy SE, Riquino MR (2017). Pain, hedonic regulation, and opioid misuse: modulation of momentary experience by mindfulness-oriented recovery enhancement in opioid-treated chronic pain patients. Drug Alcohol Depend.

[62] Manchikanti L, Giordano J, Boswell MV, Fellows B, Pampati V (2007). Psychological factors as predictors of opioid abuse and illicity drug use in chronic pain patients. J Opioid Manag.

[63] Hser Y-I, Mooney LJ, Saxon AJ, Miotto K, Bell DS, Huang D (2017). Chronic pain among patients with opioid use disorder: results from electronic health records data. J Subst Abus Treat.

[64] Hassett AL, Aquino JK, Ilgen MA (2014). The risk of suicide mortality in chronic pain patients. Curr Pain Headache Rep.

[65] Ilgen MA, Kleinberg F, Ignacio RV, Bohnert ASB, Valenstein M, McCarthy JF (2013). Noncancer pain conditions and risk of suicide. JAMA Psychiatry.

[66] Winer ES, Drapeau CW, Veilleux JC, Nadorff MR (2016). The association between anhedonia, suicidal ideation, and suicide attempts in a large student sample. Arch Suicide Res.

[67] Winer ES, Nadorff MR, Ellis TE, Allen JG, Herrera S, Salem T (2014). Anhedonia predicts suicidal ideation in a large psychiatric inpatient sample. Psychiatry Res.

[68] Zielinski MJ, Veilleux JC, Winer ES, Nadorff MR (2017). A short-term longitudinal examination of the relations between depression, anhedonia, and self-injurious thoughts and behaviors in adults with a history of self-injury. Compr Psychiatry.

[69] Garland EL, Riquino MR, Priddy SE, Bryan CJ (2017). Suicidal ideation is associated with individual differences in prescription opioid craving and cue-reactivity among chronic pain patients. J Addict Dis.

[70] Bentley KH, Nock MK, Barlow DH (2014). The four-function model of nonsuicidal self-injury: key directions for future research. Clin Psychol Sci..

[71] Gratz KL, Chapman AL, Dixon-Gordon KL, Tull MT (2016). Exploring the association of deliberate self-harm with emotional relief using a novel implicit association test. Personal Disord.

[72] Ehde DM, Dillworth TM, Turner JA (2014). Cognitive-behavioral therapy for individuals with chronic pain: efficacy, innovations, and directions for research. Am Psychol.

[73] Garland EL (2013). Mindfulness-oriented recovery enhancement: reclaiming a meaningful life from addiction, stress, and pain. Washington. D.C.: NASW press.

[74] Garland EL (2016). Restructuring reward processing with mindfulness-oriented recovery enhancement: novel therapeutic mechanisms to remediate hedonic dysregulation in addiction, stress, and pain. Ann N Y Acad Sci.

[75] Garland EL, Manusov EG, Froeliger B, Kelly A, Williams JM, Howard MO (2014). Mindfulness-oriented recovery enhancement for chronic pain and prescription opioid misuse: results from an early-stage randomized controlled trial. J Consult Clin Psychol.

[76] Davidson RJ (2010). Empirical explorations of mindfulness: conceptual and methodological conundrums. Emot Wash DC.

[77] Garland EL, Froeliger B, Howard MO. Mindfulness training targets neurocognitive mechanisms of addiction at the attention-appraisal-emotion interface. Front Psychiatry. 2014;4(173) 10.3389/fpsyt.2013.00173 PMC3887509 24454293

[78] Vago DR, Silbersweig DA. Self-awareness, self-regulation, and self-transcendence (S-ART): a framework for understanding the neurobiological mechanisms of mindfulness. Front Hum Neurosci. 2012;6(296) 10.3389/fnhum.2012.00296 PMC3480633 23112770

[79] Tomlinson E, Yousaf O, Vittersø A, Jones L. Dispositional mindfulness and psychological health: a systematic review. Mindfulness. 2017;(9):23–43. 10.1007/s12671-017-0762-6 PMC5770488 29387263

[80] Garland EL, Hanley AW, Thomas EA, Knoll P, Ferraro J. Low dispositional mindfulness predicts self-medication of negative emotion with prescroption opioids. J Addict Med. 2015;9(1):61–7. 10.1097/ADM.0000000000000090 PMC4310788 25469652

[81] Froeliger B, Garland EL, Kozink RV, Modlin LA, Chen NK, McClernon FJ, et al. Meditation-state functional connectivity (msFC): strengthening of the dorsal attention network and beyond. Evid Based Complement Alternat Med. 2012;2012:680407. 10.1155/2012/680407 PMC3320106 22536289

[82] Kang D, Jo HJ, Kim SH, Jung Y, Choi C (2013). The effect of meditation on brain structure: cortical thickness mapping and diffusion tensor imaging. Soc Cogn Affect Neurosci.

[83] Hölzel BK, Lazar SW, Gard T, Schuman-Olivier Z, Vago DR, Ott U (2011). How does mindfulness meditation work? Proposing mechanisms of action from a conceptual and neural perspective. Perspect Psychol Sci.

[84] Chambers R, Gullone E, Allen NB (2009). Mindful emotion regulation: an integrative review. Clin Psychol Rev.

[85] Garland EL, Gaylord SA, Palsson O, Faurot K, Mann JD, Whitehead WE (2012). Therapeutic mechanisms of a mindfulness-based treatment for IBS: effects on visceral sensitivity, catastrophizing, and affective processing of pain sensations. J Behav Med.

[86] Zeidan F, Martucci K, Kraft R, Gordon N, McHaffie J, Coghill R (2011). Brain mechanisms supporting modulation of pain by mindfulness meditation. J Neurosci.

[87] Zeidan F, Martucci KT, Kraft RA, McHaffie JG, Coghill RC (2014). Neural correlates of mindfulness meditation-related anxiety relief. Soc Cogn Affect Neurosci.

[88] Lazarus R, Folkman S. Stress, Appraisal, and coping. New York: Springer; 1984.

[89] Doré BP, Boccagno C, Burr D, Hubbard A, Long K, Weber J (2016). Finding positive meaning in negative experiences engages ventral striatal and ventromedial prefrontal regions associated with reward valuation. J Cogn Neurosci.

[90] Jensen KB, Kosek E, Wicksell R, Kemani M, Olsson G, Merle J (2012). Cognitive behavioral therapy increases pain-activation of the prefrontal cortex in patients with fibromyalgia. Pain.

[91] Woo C-W, Roy M, Buhle JT, Wager TD (2015). Distinct brain systems mediate the effects of nociceptive input and self-regulation on pain. PLoS Biol.

[92] Garland EL, Farb NA, Goldin P, Fredrickson BL (2015). Mindfulness broadens awareness and builds eudaimonic meaning: a process model of mindful positive emotion regulation. Psychol Inq.

[93] Goldin PR, Morrison A, Jazaieri H, Brozovich F, Heimberg R, Gross JJ (2016). Group CBT versus MBSR for social anxiety disorder: a randomized controlled trial. J Consult Clin Psychol.

[94] Garland EL, Roberts-Lewis A, Tronnier C, Graves R, Kelley K. Mindfulness-oriented recovery enhancement versus CBT for co-occurring substance dependence, traumatic stress, and psychiatric disorders: proximal outcomes from a pragmatic randomized trial. Behav Res Ther. 2016;77:7–16. 10.1016/j.brat.2015.11.012 PMC4752876 26701171

[95] Garland EL, Froeliger B, Howard MO. Effects of mindfulness-oriented recovery enhancement on reward responsiveness and opioid cue-reactivity. Psychopharmacology. 2014;231(16):3229–38. 10.1007/s00213-014-3504-7 PMC4111972 24595503

[96] Garland EL, Howard MO, Zubieta J-K, Froeliger B (2017). Restructuring hedonic dysregulation in chronic pain and prescription opioid misuse: effects of mindfulness-oriented recovery enhancement on responsiveness to drug cues and natural rewards. Psychother Psychosom.

[97] Garland EL, Froeliger B, Howard MO (2015). Neurophysiological evidence for remediation of reward processing deficits in chronic pain and opioid misuse following treatment with mindfulness-oriented recovery enhancement: exploratory ERP findings from a pilot RCT. J Behav Med.

[98] Froeliger B, Mathew AR, McConnell PA, Eichberg C, Saladin ME, Carpenter MJ (2017). Restructuring reward mechanisms in nicotine addiction: a pilot fMRI study of mindfulness-oriented recovery enhancement for cigarette smokers. Evid-Based Complement Altern Med ECAM.

[99] Finan PH, Garland EL (2015). The role of positive affect in pain and its treatment. Clin J Pain.

[100] Becker S, Wiebke G, Pomares F, Wager TD, Schweinhardt P (2017). Orbitofrontal cortex mediates pain inhibition by monetary reward. Soc Cogn Affect Neurosci.

[101] Garland EL, Howard MO. Enhancing natural reward responsiveness among opioid users predicts chronic pain relief: EEG analysis from a trial of mindfulness-oriented recovery enhancement. J Soc Soc Work Res. 2018; in press 10.1086/697685 PMC6402811 30854168

